# The management of incidental appendiceal mucinous neoplasm encountered during appendectomy: A single center experience.

**DOI:** 10.1097/MD.0000000000051261

**Published:** 2026-10-23

**Authors:** Chong Wang, Zhongwu Ma, Puyu Yao, Lubiao An, Guanjun Shi, Haipeng Zhou, Pu Zhang, Ruiqing Ma

**Affiliations:** a Department of Myxoma, Aerospace Center Hospital, Beijing 100049, China; b Wenzhou Central Hospital, The Dingli Clinical Institute of Wenzhou Medical University, Wenzhou 325000, Zhejiang Province, China; c Department of Anesthesiology, Aerospace Center Hospital, Beijing, 100049, China.

**Keywords:** cytoreductive surgery (CRS), incidental appendiceal mucinous neoplasm (IAMN), pathologic subtype, peritoneal pseudomyxoma (PMP), right hemicolectomy (RH)

## Abstract

The management of incidental appendiceal mucinous neoplasm (IAMN) was confusing with difficult decision-making. This study was to investigate the association of variables from the initial operation to cytoreductive surgery with overall survival (OS). One hundred thirty patients of IAMN treated at Aerospace Center Hospital were retrospectively analyzed from January 2014 to December 2020. A total of 82 IAMN patients who underwent reoperation with cytoreductive surgery (CRS) and had available data on the date of their initial operation was included. Of these 82 IAMN patients, the mean time from the initial operation to CRS was 19 months (1–146 months). The 5-year OS rate was 69.8%, and the median OS was 39 months (1–73 months). The patients who underwent CRS within 6 months had a higher radical rate (63.3% vs 36.7%) with lower tumor burden and better survival. No significant difference was found in patients with right hemicolectomy or prior systemic chemotherapy. Multivariate analysis identified the completeness of cytoreduction score and the pathologic subtype of peritoneal pseudomyxoma (PMP) as independent prognostic factors. Extended surgery and systematic chemotherapy are not suitable for IAMN patients. It is more important to identify the pathology and accept standard therapy in academic medical centers within 6 months.

## 1. Introduction

Appendiceal mucinous neoplasm (AMN) is a rare tumor with a low proportion in appendectomy specimens.^[1,2]^ These tumors may infiltrate the serosa of the appendix and spread to the peritoneal cavity, resulting in a syndrome named pseudomyxoma peritoneum (PMP).^[3,4]^ In 2020, international experts from Peritoneal Surface Oncology Group International developed a uniform pathological classification of PMP and non-carcinoid epithelial neoplasia of the appendix.^[5]^ The categories include serrated polyplow, low grade appendiceal mucinous neoplasm (LAMN), high grade appendiceal mucinous neoplasm (HAMN), and appendiceal mucinous adenocarcinoma (AMA, with or without signet ring cells). The classification of PMP includes acellular mucin, disseminated peritoneal adenocarcinoma (DPAM), peritoneal mucinous carcinomatosis (PMCA), and peritoneal mucinous carcinomatosis with signet ring cells (PMCA-S). AMN is usually unexpectedly discovered at any elective or emergency abdominal surgery with difficult decision-making, especially when appendectomy was performed because of appendicitis with or without PMP.^[1,2]^ The management of incidental appendiceal mucinous neoplasm (IAMN) remains challenging. Patients with simultaneous or heterochronic PMP usually accepted repetitive operations until receiving cytoreductive surgery (CRS) combined with hyperthermic intraperitoneal chemotherapy (HIPEC), which was considered the standard treatment for PMP.^[6,7]^ Due to the ignorance of 2 rare diseases and informal treatments, many patients received informal treatment or missed the best treatment period. This study was to investigate the association of variables from the initial operation to cytoreductive surgery with overall survival.

## 2. Materials and methods

The protocol for this study was conducted in accordance with relevant laws and institutional guidelines and approved by the Ethics committee of the Aerospace Center Hospital, Beijing, China (No. 2022-061). The written informed consent was obtained for all patients.

### 2.1. Patients

We retrospectively analyzed the patients who presented to at Aerospace Center Hospital, Beijing, China, from 01/01/2014 to 31/12/2020. No information that could identify individual participants during or after data collection. The study included patients in whom AMN was incidentally discovered during appendectomy performed for appendicitis and who had not undergone prior CRS. The resectability of surgery was evaluated according to enhanced CT of the abdominal and pelvic cavity. Indications for CRS included: the absence of obvious abdominal or pelvic lesions but with evidence of appendix perforation or serosal involvement; or the presence of definitive abdominal lesions. The pathological subtype was confirmed by at least 2 experienced pathologists. Laparoscopic exploration was performed for patients with limited disease and low tumor burden, and an open CRS was selected when laparoscopic CRS was difficult. Open CRS was directly selected for patients with high tumor burden. All patients were followed up every 3–6 months after CRS, and the end of the follow-up date was December 2021. A flowchart outlining the study population is presented in Figure 1.

**Figure 1.**
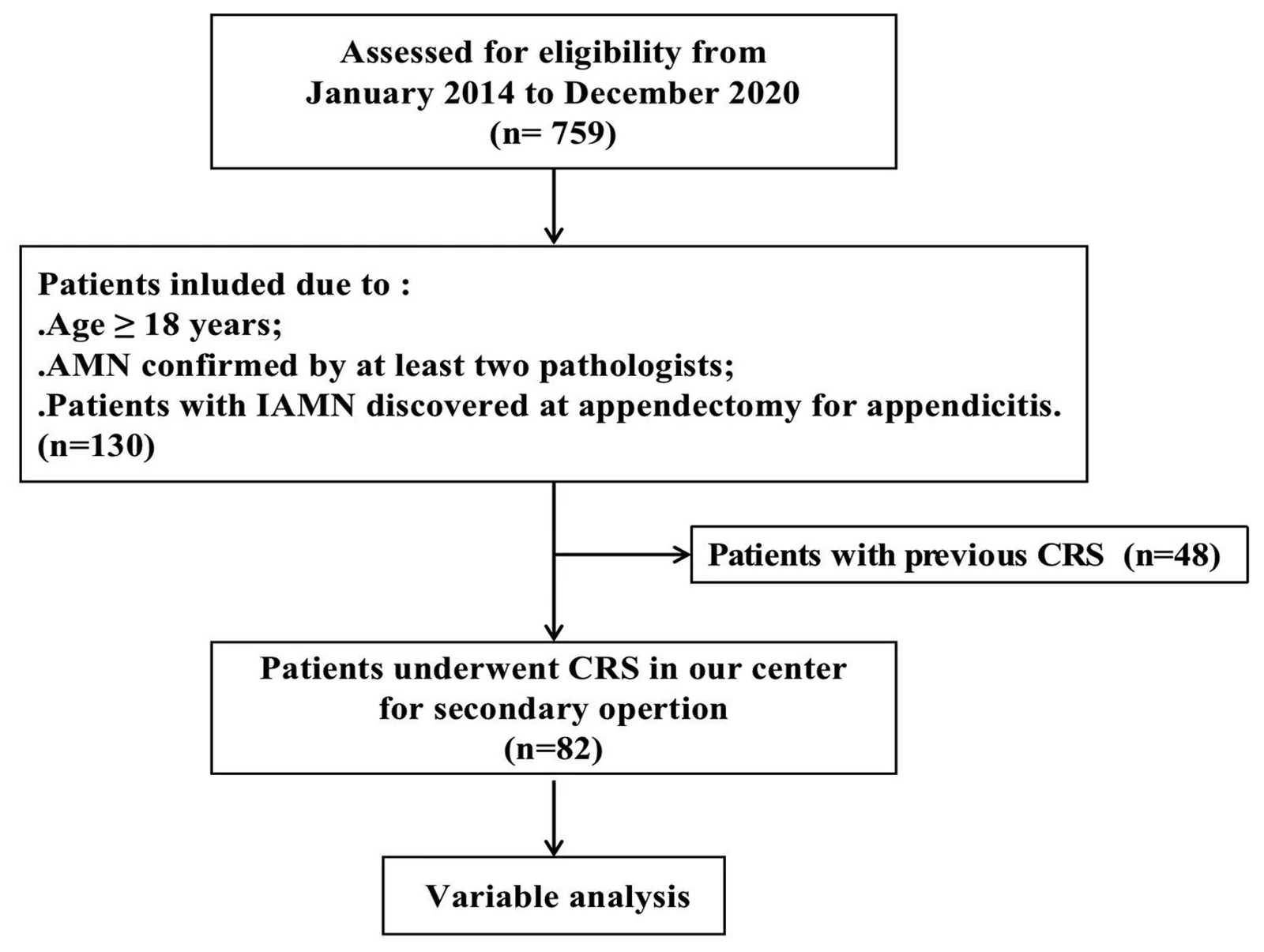
The flowchart of patient screening. IAMN = incidental appendiceal mucinous neoplasm, CRS = cytoreductive surgery.

### 2.2. Data collection

The demographic data, perioperative clinical and pathological parameters of patients were collected, including age, sex, intraoperative findings (specifically, the presence or absence of appendix perforation), prior surgical score (PSS), prior systemic chemotherapy (PSC), peritoneal cancer index (PCI), completeness of cytoreduction (CCR) score, and HIPEC. PSS ranged from 0 to 3: 0: biopsy only; 1: minimal prior dissection with only one abdominal region dissected; 2: 2 to 5 regions dissected; 3: >5 regions dissected. The PCI score was evaluated as described by Sugarbaker,^[8]^ which ranges from 0 to 39. Complications were graded using the Clavien-Dindo classification. Postoperative deaths occurring within 30 days (n = 0) were excluded from the survival analysis.

### 2.3. Time interval groups and overall survival

The time interval from the date of the initial operation to the date of CRS in our center was calculated for all patients. Patients were stratified into 2 groups according to clinical pragmatism and median time interval: less than 6 months, and more than 6 months. The clinical variables of each group were compared. The overall survival (OS) was calculated from the CRS in our center to the last follow-up or death date.

### 2.4. Operation

Cytoreductive surgery: the median abdominal incision for patients who underwent open CRS, and 4 trocars were placed in the subumbilical (10 mm), right upper abdomen (5 mm), left upper abdomen (10 mm), and left lower abdomen (5mm) for patients who underwent laparoscopic CRS. The intraoperative peritoneal cancer index (PCI) was evaluated. Peritonectomy combined with organ resection was performed to remove all visible tumors as much as possible, as described by Sugarbaker.^[9]^ Ovariectomy and greater omentum resection are applicable to patients without obvious lesions. A CCR score was recorded after CRS, and the standard is as follows^[9]^: CCR0: no visible residue; residual tumor: CCR1: <2.5 mm, CCR2: 2.5 mm, 2.5 cm, CCR3: >2.5 cm.

HIPEC: HIPEC was performed after CRS and before digestive tract reconstruction with a closed procedure. Two inflow and two outflow catheters were placed in the abdomen and connected to a hyperthermia chemotherapy perfusion machine (Jilin Minda Company products, RHL - 2000B) with a 60 minutes treatment time. The circulation speed was controlled at 800 ~ 1000 ml/min, and the outlet temperature was controlled at 41°C when the inlet temperature was controlled at 43.5°C. Cisplatin 60–80 mg was used with 3500~4000ml physiological saline. The HIPEC was performed for patients with CCR 0 to 3.

### 2.5. Statistical analysis

SPSS 25.0 (IBM Corporation, Armonk, NY, USA) and R 4.1.2 (http://www.r-project.org) were used for statistical analysis. The continuous variables were expressed as median (interquartile ranges, IQR) and analyzed by the Mann–Whitney U test. The categorical variables were presented as frequency with percentage [n (%)] and compared with chi-square or Fisher’s exact test. The Kaplan–Meier method was applied to compare survival curves of predictive factors, and the significance of these differences was tested by Log-rank test. A multivariable Cox proportional hazards model was constructed upon the statistically significant univariate analysis. The significance level for all statistical tests was set at 0.05.

## 3. Results

### 3.1. Patient characteristics

A total of 130 patients from January 2014 to December 2020 were analyzed. Forty-eight patients were excluded. Eighty-two patients underwent second operation with CRS in our center. All these patients only underwent appendectomy at the initial operation. Among these, 67 patients underwent open CRS and 15 underwent laparoscopic CRS. All patient diagnosed as PMP. The average age was 54 ± 12 years, with 40 men and 42 women. Other characteristics are shown in Table 1.

**Table 1:** The clinical characteristics of patients.

Variable	Patients underwent CRS (n = 82)
Age, y,median (IQR)	57 (47.0~67.0)
Sex, n (%), Women/Men	42 (51.2)/40 (48.8)
PSS, n (%), 0–1/2–3	50 (61.0)/32 (39.0)
PSC, n (%), No/Yes	67 (81.7)/15 (18.3)
Perforation, No/Yes	66 (80.5)/16 (19.5)
Operation, n (%),Open/Laparoscope	70 (85.4)/12 (14.6)
PCI, median (IQR)	10 (0.75~29.0)
HIPEC, n (%), No/Yes	4 (4.9)/78 (95.1)
CCR, n (%), 0–1/2–3	59 (72.0)/23 (28.0)
RH, n (%), No/Yes	27 (32.9)/55 (67.1)
Pathologic subtype of AMN, n (%), LAMN/HAMN/AMA	53 (64.6)/3 (3.7)/26 (31.7)
Pathologic subtype of PMP, n (%), DPAM/PMCA/PMCA-S	54 (65.9)/21 (25.6)/7 (8.5)
Complication, n (%), No/Yes	48 (58.5)/34 (41.5)
Time interval (months), n (%), ≤6/>6	41 (50.0)/41 (50.0)

AMA = appendiceal mucinous adenocarcinoma, CCR = completeness of cytoreduction, DPAM = disseminated peritoneal adenocarcinoma, HAMN = high-grade appendiceal mucinous neoplasm, HIPEC = hyperthermic intraperitoneal chemotherapy, LAMN = low-grade appendiceal mucinous neoplasm, PCI = peritoneal cancer index, PMCA = peritoneal mucinous carcinomatosis, PMCA-S = peritoneal mucinous carcinomatosis with signet ring cells, PMP = peritoneal pseudomyxoma, PSC = prior systemic chemotherapy, PSS = prior surgical score, RH = right hemicolectomy.

### 3.2. The results of two time interval group

Among the entire cohort, the median time to CRS was 6.5 months, resulting in 2 groups of 41 patients each (≤ 6 vs > 6 months). Comparative analyses of clinical factors between the 2 groups are shown in Table 2. Patients who underwent CRS within 6 months achieved a significantly higher complete cytoreduction (CCR-0/1) rate (63.3% vs 36.7%) and had a lower overall tumor burden. There was no difference in median OS between the 2 groups.

**Table 2:** Clinical features of patients with IAMN by time interval group.

Variable	No.		*P* value
	≤6 months	>6months	
Age, y, median (IQR)	53 (46.5~61.5)	60 (47.5~63.5)	.181
Sex, n			
Men	25	15	
Women	16	26	.027
PSS, n			
0–1	32	18	
2–3	9	23	.002
PSC, n			
No	38	29	
Yes	3	12	.010
Perforation, n			
No	30	36	
Yes	11	5	.095
Operation, n			
Open	31	39	
Laparoscope	10	2	.012
PCI, median (IQR)	1 (0~5.5)	29 (16~31)	<.001
HIPEC, n			
No	0	4	
Yes	41	37	.116
CCR, n			
0–1	38	22	
2–3	3	19	<.001
RH, n			
No	14	13	
Yes	27	28	.814
Pathologic subtype of AMN, n			
LAMN	31	22	
HAMN	0	3	
AMA	10	16	.059
Pathologic subtype of PMP, n			
DPAM	34	20	
PMCA	5	16	
PMCA-S	2	5	.005
Complication, n			
No	32	16	
Yes	9	25	<.001
OS, months, median (IQR)	35 (19~36.5)	33 (14.5~51.5)	.519

AMA = appendiceal mucinous adenocarcinoma, CCR = completeness of cytoreduction, DPAM = disseminated peritoneal adenocarcinoma, HAMN = high-grade appendiceal mucinous neoplasm, HIPEC = hyperthermic intraperitoneal chemotherapy, LAMN = low grade appendiceal mucinous neoplasm, PCI = peritoneal cancer index, PMCA = peritoneal mucinous carcinomatosis, PMCA-S = peritoneal mucinous carcinomatosis with signet ring cells, PMP = peritoneal pseudomyxoma, PSC = prior systemic chemotherapy, PSS = prior surgical score, RH = right hemicolectomy.

### 3.3. The results of OS

For 82 patients who underwent CRS, the median duration of follow-up was 35 months (1–71 months). By the last follow-up on December 2021, 18 patients had died, the 5-year OS rate was 69.8%. No one received RH before in these 82 patients. After CRS in our center, 55 patients received RH, however, this procedure conferred no significant OS benefit compared to patients who did not receive RH. Subgroup analyses based on pathology also revealed no significant survival benefit associated with RH, either for patients with LAMN or for those with HAMN/AMA (Fig. 2A–C). Analysis of the time interval to CRS demonstrated that patients undergoing surgery within 6 months had significantly better survival outcomes (Fig. 3). Conversely, patients who had received prior systemic chemotherapy (PSC) exhibited poorer survival (Fig. 4). After multivariate analysis, the pathologic subtype of PMP and completeness of cytoreduction score were selected as independent predictors of prognosis for IAMN after CRS (Table 3).

**Table 3:** Univariate and multivariate analyses for prognostic factors affecting overall survival after CRS.

Variables	No.	Univariate analysis		Multivariate analysis	
		Hazard ratio(95%CI)	*P*	Hazard ratio(95%CI)	*P*
Age(years)					
<55	38	1	–	–	–
≥55	44	1.403 (0.543~3.622)	.485	–	–
Sex, n					
Women	42	1		–	–
Men	40	0.955 (0.395~2.307)	.918	–	–
PSS, n					
0–1	50	1	–	–	–
2–3	32	3.686 (1.382~9.832)	.009	–	–
PSC, n					
No	67	1	–	–	–
Yes	15	3.332 (1.289~8.614)	.013	–	–
Perforation, n					
No	66	1	–	–	–
Yes	16	0.845 (0.245~2.922)	.791	–	–
Operation, n					
Open	70	1	–	–	–
Laparoscope	12	0.039 (0.001~21.427)	.314	–	–
PCI, n					
<20	47	1	–	–	–
≥20	35	12.312 (2.827~53.623)	.001	–	–
HIPEC, n					
No	4	1	–	–	–
Yes	78	0.021 (0.005~0.088)	<.001	–	–
CCR, n					
0/1	60	1	–	1	–
2/3	22	8.931 (3.178~25.093)	<.001	4.463 (1.136~17.533)	.032
RH, n					
No	27	1	–	–	–
Yes	55	1.529 (0.502~4.658)	.455	–	–
Pathologic subtype of AMN, n					
LAMN	53	1			
HAMN	3	2.676 (0.328~21.844)	.358	–	–
AMN	26	3.293 (1.253~8.659)	.016	–	–
Pathologic subtype of PMP, n					
DPAM	54	1	–	1	–
PMCA	21	5.943 (1.787~19.764)	.004	3.922 (1.020~15.087)	.047
PMCA-S	7	22.967 (6.298~83.762)	<.001	11.612 (1.858~72.593)	.009
Complication, n					
No	48	1	–	–	–
Yes	34	25.495 (3.392~191.651)	.002	–	–
Time interval (months)					
≤6	41	1	–	–	–
>6	41	5.246 (1.518~18.128)	.009	–	–

AMA = appendiceal mucinous adenocarcinoma, CCR = completeness of cytoreduction, CRS = cytoreductive surgery, DPAM = disseminated peritoneal adenocarcinoma, HAMN = high grade appendiceal mucinous neoplasm, HIPEC = hyperthermic intraperitoneal chemotherapy, LAMN = low low-grade appendiceal mucinous neoplasm, PCI = peritoneal cancer index, PMCA = peritoneal mucinous carcinomatosis, PMCA-S = peritoneal mucinous carcinomatosis with signet ring cells, PMP = peritoneal pseudomyxoma, PSC = prior systemic chemotherapy, PSS = prior surgical score, RH = right hemicolectomy.

**Figure 2.**
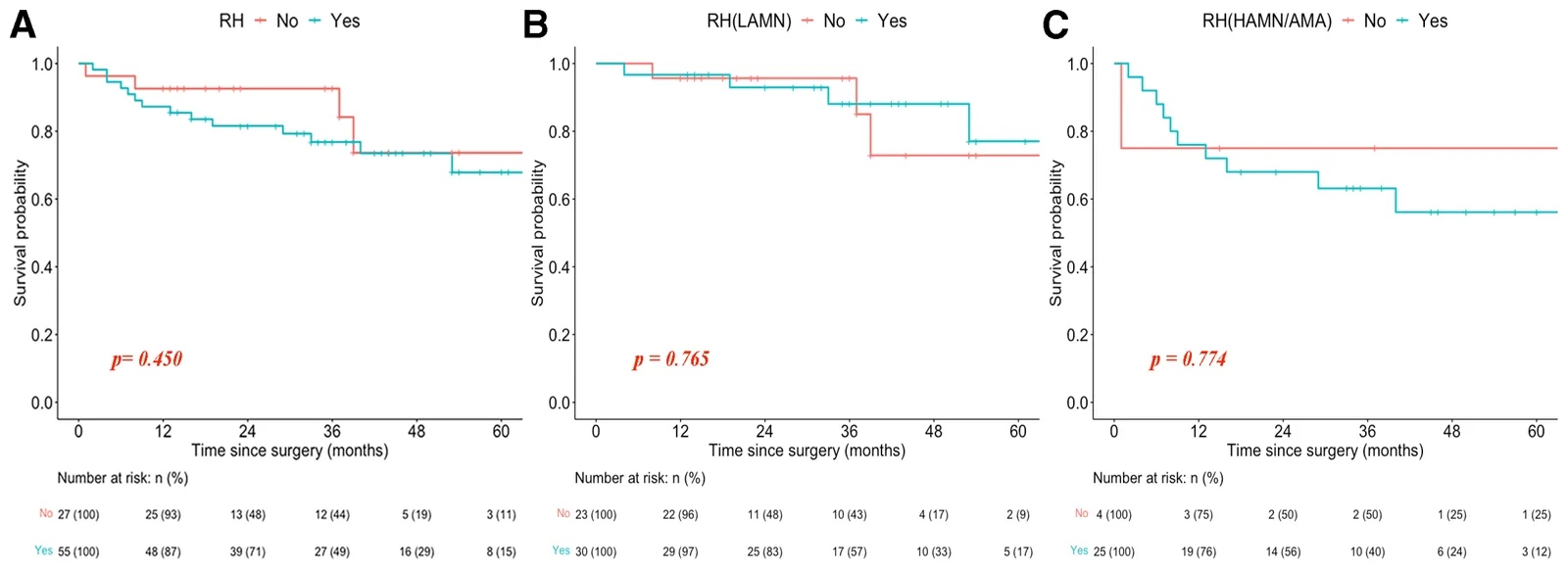
Kaplan–Meier curves show the survival difference in RH (A) and RH in pathologic subtype (B and C). No survival benefit for OS was found compared to patients with RH, even in the pathological subgroups between DPAM and PMCA/PMCA-S. RH = right hemicolectomy, DPAM = disseminated peritoneal adenocarcinoma, PMCA = peritoneal mucinous carcinomatosis, PMCA-S = peritoneal mucinous carcinomatosis with signet ring cells.

**Figure 3.**
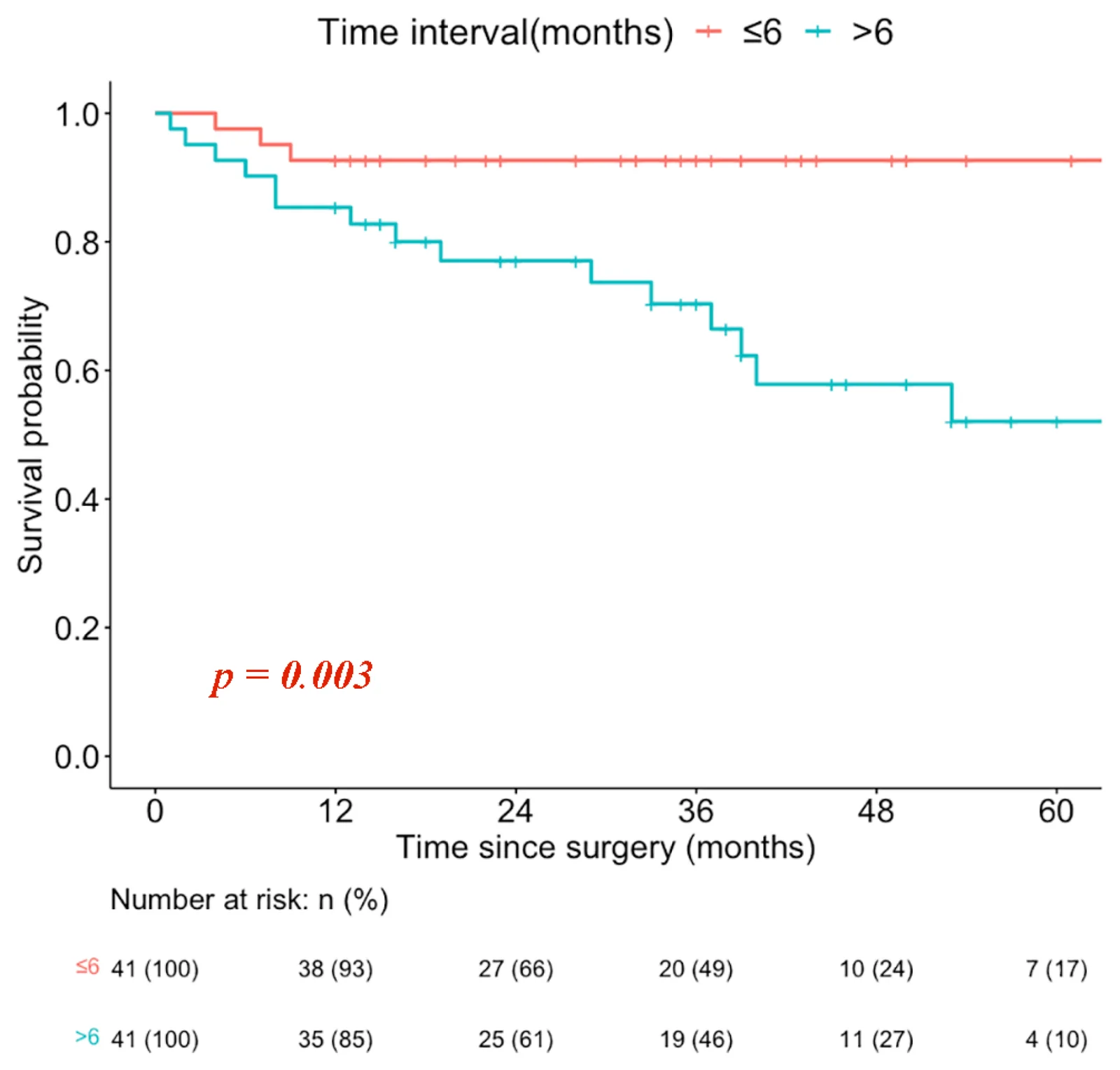
Kaplan–Meier curves show the survival difference in time interval. The patients who underwent CRS within 6 months had better survival.

**Figure 4.**
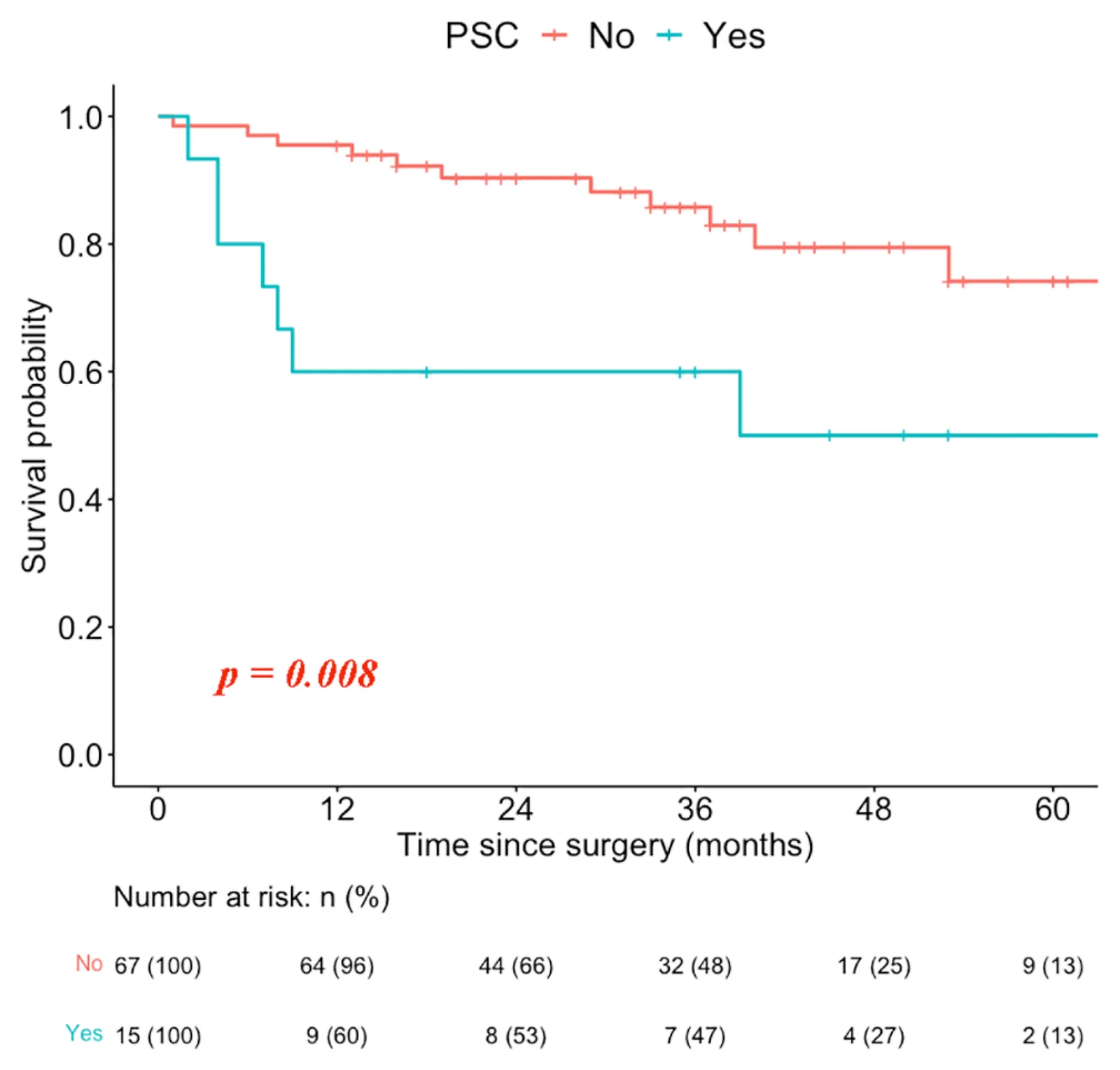
Kaplan–Meier curves show the survival difference in PSC. The patients who underwent PSC had poor survival. PSC = prior systemic chemotherapy.

## 4. Discussion

Appendiceal mucinous neoplasm belongs to epithelial tumors. Unlike their non-epithelial counterparts, they often lack classic malignant features such as lymphovascular invasion or distant metastasis. When accompanied with appendiceal perforation, PMP generally exists with slow but relentless growth, resulting in a fatal outcome because of progressive obstruction.^[10,11]^ An appendiceal mucinous neoplasm is frequently unexpectedly found in operations for appendicitis, presenting significant therapeutic dilemmas for surgeons. Historically, the RH was routinely performed for appendiceal epithelial neoplasms. But from the existing research, the RH is maybe not suitable for all types of appendiceal epithelial tumors. Appendectomy only was recommended for LAMN because of lower lymph node involvement.^[4]^ However, a clear margin should be obtained with a lower risk for recurrence.^[12]^ Interestingly, surgeons performed an appendectomy with ileocecectomy over RH to avoid a positive appendiceal margin.^[13]^ Whether a difference exists in prognosis between the 2 operations is still unclear.

Current debate primarily centers on the management of HAMN and AMA, referred to as PMCA or PMCA-S. Murphy et al concluded in their systematic review that the RH provided a longer OS than an appendicectomy alone in non-perforated appendices. But in perforated appendices or with PMP, the advice of RH was reserved.^[14]^ The study reported by professor Sugarbaker indicated that RH was recommended in high-grade PMCA with a high lymph node invasion rate,^[15]^ while other studies have found no survival benefit from RH in node-positive or metastatic AMA.^[16]^ Research on the role of RH in low-grade AMA similarly reported no improvement in OS, suggesting its use should be reserved for cases with positive margins following initial appendectomy.^[17]^ In 2020, the clinical practice guidelines for appendiceal tumors and PMP conducted by PSOGI united with European Rare Cancer (EURACAN) proposed detailed suggestions on RH for various pathological types of IAMN, the RH should strongly not be considered for LAMN, and the RH should be performed for HAMN and AMA with or without PMP.^[6]^ Consistent with these evolving perspectives, our study found no OS benefit from RH in any pathological subgroup. At our institution, ileocecectomy or RH is performed for LAMN patients to secure clear margins, while RH is routinely performed for HAMN and AMA due to their potential for lymphatic metastasis, irrespective of PMP presence.

The CRS combined with HIPEC was an internationally recognized standard treatment for PMP despite the lack of randomized data, significantly improving the patients’ long-term survival rate. Of these 82 patients who received CRS in our center, the 5-year OS rate was 69.8%. The completeness of cytoreduction (CCR) is an important index to evaluate the CRS with poor prognosis in patients who achieved CCR 2-3 compared to CCR 0-1.^[18,19]^ In this study, 59 (72.0%) patients underwent CCR 0/1 in our center with a better OS. The proportion of pathological subgroups was 65.9% (DPAM, n = 54), 25.6% (PMCA, n = 21) and 8.5% (PMCA-S, n = 7), respectively. A cohort study by Kusamura et al demonstrated improved OS with HIPEC in patients with CCR 0-3.^[20]^ However, its efficacy in patients with CCR 2/3 remains unproven, likely due to limited drug penetration into larger tumor deposits.^[21]^ It is emphasized that HIPEC should only follow adequate cytoreduction, and this combined modality represents the first-line treatment for IAMN.

The subtypes of PMP were mostly associated with AMN. The discordance between the pathological grading of the AMN and PMP also has been reported.^[22,23]^ Notably, one analysis found that patient OS correlates more strongly with the peritoneal disease pathology than with the grade of the primary appendiceal tumor.^[24]^ A significant impact on the prognosis of the pathological subtypes of PMP has been confirmed.^[25,26]^ Our multivariate analysis corroborates this, identifying the PMP pathological subtype as an independent risk factor for OS, underscoring its critical role in determining prognosis for IAMN patients with PMP.

Adjuvant systemic chemotherapy was an essential treatment for malignant tumors. Unfortunately, the impact of systemic chemotherapy on AMN and PMP was still unclear. Asare et al conducted a large cohort study to discuss the effect of systemic chemotherapy for the 5971 patients of AMA, and the result showed that no benefit from systemic chemotherapy was found in patients with well-differentiated stage IV, and the OS was significantly increased in patients with moderately and poorly differentiated AMA.^[27]^ Blackham et al Reported that patients with high-grade appendiceal PMP had a longer PFS after chemotherapy, although without an obvious survival advantage.^[28]^ Conversely, another study found no association between chemotherapy and OS in stage IV low-grade AMA.^[29]^ A cohort study from our center found preoperational systemic chemotherapy did not benefit for appendiceal PMP.^[30]^ In this study, we also found the patients who accepted systemic chemotherapy had significantly poor survival before CRS. Hence, systemic chemotherapy was maybe not undesirable for IAMN. The multicenter prospective randomized controlled studies were urgently needed for AMN and PMP.

Due to the slow progression of PMP, some patients can achieve long-term survival even without any intervention. Theoretically, Theoretically, earlier intervention should yield better outcomes. The optimal time interval for CRS after the first operation for both patient selection and long-term survival is not known. In this study, we compared the clinical variables of each group which set at 6 months, and the effect of interval time on OS was also calculated. The patients who underwent CRS within 6 months had a higher radical rate (63.3% vs 36.7%) with lower tumor burden. Although the median OS was not significantly different, survival curves favored the early CRS group (Fig. 3). Thus, prompt referral to academic medical centers for standard therapy is crucial. Further investigation is required to determine the definitive optimal time interval, and our center is initiating a multicenter study to address this question.

There are several limitations in this study. Firstly, this cohort study was from a single center with a small clinical database and a short follow-up period. However, as the academic medical centers, the clinical characteristics were like international reporting.^[4]^ Secondly, the first treatment of these 82 patients was not in our center, for which data bias may exist. To control this bias, all these patients were required to provide detailed treatment records, including pathological and surgical records. Thirdly, the cases of AMN found accidentally for other reasons were not included in this study because of the small sample.

## 5. Conclusion

Extended surgery and systematic chemotherapy are not suitable for IAMN patients. It is more important to identify the pathology and accept standard therapy in academic medical centers within 6 months.

Supplemental Digital Content “Two-Sentence Article Summary” is available for this article

## Author contributions

**Formal analysis:** Chong Wang, Lubiao An, Pu Zhang.

**Writing – original draft:** Chong Wang, Zhongwu Ma, Puyu Yao.

**Conceptualization:** Zhongwu Ma.

**Data curation:** Puyu Yao, Guanjun Shi, Haipeng Zhou.

**Methodology:** Lubiao An, Guanjun Shi, Haipeng Zhou.

**Investigation:** Guanjun Shi.

**Writing – review & editing:** Ruiqing Ma.


