# Supplementary material for: The management of incidental appendiceal mucinous neoplasm encountered during appendectomy: A single center experience
Source: Medicine (Baltimore). 2026 Oct 23;105(43):e51261. doi: 10.1097/MD.0000000000051261 (PMC13706343; doi:10.1097/MD.0000000000051261)
Supplement: Supplementary file 1 [file medi-105-e51261-s001.docx]

This was a control study on the accidental discovery of appendiceal mucinous tumors or with peritoneal pseudomucinous tumors during appendectomy for appendicitis. The final results showed that extended surgery and systematic chemotherapy are not suitable for IAMN patients. It is more important to identify the pathology and accept standard therapy in academic medical centers within 6 months.
